# Structural characteristics and interspecific relationship changes during the succession process of the Fagaceae community in a subtropical forest

**DOI:** 10.3389/fpls.2025.1489090

**Published:** 2025-03-27

**Authors:** Yuxin Ying, Zhibin Wu, Yao Yan, Xianzhen Zhou, Wei Gao

**Affiliations:** Institute of Forest Ecology and Carbon Sink Measurement, Fujian Forestry Vocational and Technical College, Nanping, Fujian, China

**Keywords:** subtropical forest, community succession, stand structure, interspecific relationship, biodiversity

## Abstract

Interspecific relationships can reflect the relevance of species in particular spatial distribution, and the degree of community adaptation under successional stages. In the past, numerous studies on subtropical forest communities primarily focused on the relationship between a specific dominant tree species or a single succession stage, lacking an understanding of interspecific relationships across different succession stages. Given this gap, this study used the method of space instead of time to study the community structure composition and interspecific relationship of trees with DBH > 1 cm in the subtropical 30-70 a Fagaceae community. The results showed that there was no significant difference in the important values of each tree species during the 30 a and 40 a of community succession. As succession advanced, the importance values of neutral and shade-tolerant species progressively escalated. The stand density reached its nadir during the mid-successional stage (50 years), concurrently achieving peak volume. Throughout succession, the diameter at breast height (DBH) distribution of sun-tolerant species conformed to an inverted J-shape, while shade-tolerant species shifted from an inverted J-shaped to a bell-shaped distribution. Positive interspecific correlations gradually intensified after 50 years, at which point both the biodiversity index and stand density reached their lowest levels, subsequently increasing as succession progressed. At the 70-year mark, a overwhelming majority (93.3%) of species pairs demonstrated no significant correlation, indicative of a more relaxed interspecific relationship. These studies show that in the early stage of community development, the community structure has not yet been finalized. With the advancement of community succession, the competition among species changes from strong to weak. In the later stage of succession, the interspecific relationship is relatively loose, and the phenomenon of seedling renewal and filling occurs in the forest, and a certain dynamic balance is maintained among various species. These results deepen the understanding of species interactions and spatial-temporal changes of community structure in different succession stages of Fagaceae communities in subtropical regions, and provide a theoretical basis for forest managers and vegetation ecological restoration in subtropical regions.

## 1 Introduction

The structural characteristics and interspecific relationships of plant communities reflect the spatial distribution and resource utilization of species, thereby characterizing the coexistence and stability of community species (Schluter, 1984; Jiang et al., 2022a). During vegetation succession, changes in plant community structure can reveal the dynamics and patterns of populations across different age classes or individuals over time, interactions within and between populations, and shed light on the future direction of population succession (Kang et al., 2014; Farahat, 2020; Xie et al., 2022). Interspecific relationship is an important quantitative and structural index in plant communities. It is a manifestation of the interaction between populations and the basis of community formation and succession (Deng et al., 2003). With the succession of plant communities, the positive or negative effects of interspecific relationships directly or indirectly affect the dynamics, species and distribution of communities (Armas and Pugnaire, 2005).By combining community structural characteristics and interspecific relationships, a better understanding of population recovery and regeneration status, as well as interspecific interactions, can be achieved, providing a theoretical basis for predicting plant community dynamics and managing community biodiversity (Liu and Zhang, 2018; Chen et al., 2022).

Community structure and species relationships are often influenced by site conditions, species composition, and developmental stages (Kim et al., 2013). For example, in the tropical area, among the main woody plants in the *Cunninghamia lanceolata* plantation community, the positive correlation preponderates over the negative correlation (Wang et al., 2024); In the subtropical region, with the natural succession of the *Loropetalum chinense* community, the interspecific relationship between the tree layer and the shrub layer gradually trends towards a positive association, and no distinct vertical structure is formed during this process (Jian et al., 2021). In the temperate zone, there are more positive connections between the dominant tree species in the tree layer of the natural secondary forest community, which enhances the stability of the community structure, and there are also negative connections among the dominant tree species in the tree layer. Shrubs invaded the ecotone between forest and grassland, and the overall association changed from positive to negative, which changed the species composition of the community (Gu et al., 2017; Lv et al., 2019; Song and Wang, 2022). The interspecific relationship of plant communities such as grasslands and wetlands in the sub-frigid zone is relatively loose, and with the increase of recovery time, the emergence of other species will lead to a decrease in the proportion of positive and negative associations, and the community structure is not stable (Gao et al., 2011; Wu et al., 2022). In summary, the study reveals that the succession of forest communities is a dynamic process. As the latitude increases, the hierarchical structure of forest communities gradually simplifies, and the competitive relationship among plants transforms from fierce to gentle. In the competitive exclusion principle, the species in the community cannot coexist for a long time, but in the competitive coexistence, it is believed that two or more organisms in a competitive relationship can coexist stably in a system. In fact, the community structure characteristics and interspecific relationships of forests are still very complex in the process of succession, and will change dynamically over time. In the past, numerous studies on subtropical forest communities primarily focused on the relationship between a specific dominant tree species or a single succession stage, lacking an understanding of interspecific relationships across different succession stages (Jian et al., 2021; Xie et al., 2022). Therefore, Exploring the changes of community structure characteristics and interspecific relationships during community succession at different succession stages of subtropical forests is helpful to provide theoretical support for better formulating scientific and reasonable forest management and protection strategies.

China possesses the world’s largest subtropical forest community, characterized by complex species composition and rich biodiversity. However, with the development of human society, the subtropical forest ecosystem in China has declined (Ma et al., 2020). As the dominant tree species in subtropical forest communities, the Fagaceae population has strong adaptability to the environment and excellent natural regeneration. It plays an important role in maintaining species diversity and community structure stability in the region (Zhao et al., 2013). Relevant studies have found that extreme weather, rising temperature, environmental pollution and other factors lead to the gradual degradation of the community structure of Fagaceae and the decrease of species diversity, which slows down the succession process and natural renewal speed (Wang et al., 2012; Wu et al., 2018; Farooq et al., 2023; Foest et al., 2024). Therefore, it is of great theoretical value to study the changes of community structure and interspecific relationship in the process of forest succession for guiding the restoration and protection of subtropical forest communities. Based on this hypothesis (1) The succession process of the subtropical Fagaceae community conforms to the theory of competitive coexistence. (2) The community structure of the subtropical Fagaceae is closely related to the interspecific relationship. (3) Moderate competition among species during succession can improve community biodiversity, which is consistent with the ‘ moderate disturbance ‘ hypothesis. In order to verify the above scientific hypothesis, this study used the method of space instead of time, selected 30 a-70 a plots under the same site conditions, and discussed the changes of community structure composition and interspecific relationship in different succession stages of subtropical Fagaceae community, which could provide theoretical basis for subtropical biodiversity conservation and ecological restoration in this area.

## 2 Materials and methods

### 2.1 Study area overview

The study plots are located in Nanping city, Fujian Province, and are characterized by a subtropical monsoon climate with an average annual temperature of 19.8°C and an annual rainfall of 1700 mm. The region spans an elevation range of 200-400 m, with red soil predominant and a pH range of 4 to 5.5. The study area is a subtropical Fagaceae community with rich species (**Table 1**). The main dominant tree species are *Quercus chungii*, *Quercus glauca*, *Castanopsis fargesii*, etc., and the associated tree species are *Loropetalum chinense*, *Symplocos sumuntia*, *Elaeocarpus decipiens*, *Camellia fraterna*, etc. The site of this study was harvested in the 1950-1990 s, and no artificial afforestation and other tending measures were carried out after harvesting. due to the natural regeneration species are native species, with a high degree of adaptability to site conditions, the main interference comes from the dominant trees by lightning or under pressure trees lack of light, found in the field of Cyclobalanopsis species have sprout regeneration ability, and in the forest has a seedling bank, and *Pinus massoniana* and *S. superba* and other species are mostly in the gap to fill with seed seedlings update.

**Table 1:** Number of woody plant families, genera and species in the forest community at different successional times.

Plot	Succession time	Family	Genus	Species
Sha Jiyang	30a	26	41	54
Sha Jiyang	40a	24	34	51
Huang Liyuan	50a	17	25	36
Shuang kenkou	70a	20	32	47

### 2.2 Plot setup and survey

**Figure 1:**
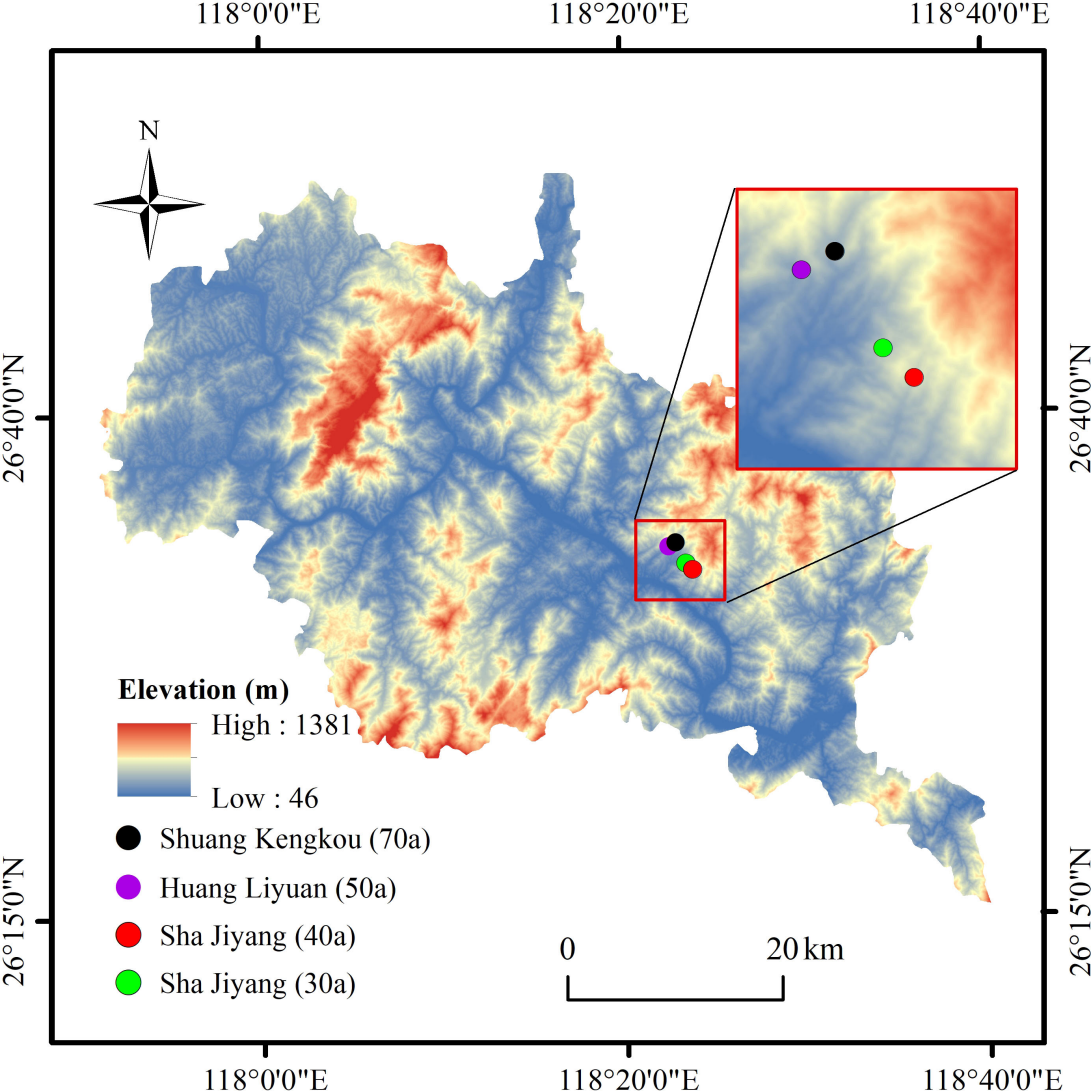
Study plot location.

In August 2020, forest plots representing different stages of succession (30, 40, 50, and 70 years) were selected in the areas of Sha Jiyang, Huang Liyuan, and Shuang Kengkou in Nanping city (**Figure 1**). The 30-year and 40-year communities in Sha Jiyang represent the early succession stage and is dominated by shrubs and small to medium-sized trees with rich species diversity. The 50-year community in Huang Liyuan represents the mid-succession stage and is characterized by a canopy dominated by large trees with a distinct dominant species. The 70-year community in Shuang Kengkou represents the late succession stage and is dominated by large trees with canopy gaps and additional species. Within each community, a 50 m × 50 m plot was established and further divided into 25 subplots of 10 m × 10 m. All trees with a diameter at breast height (DBH) greater than 1 cm within each subplot were surveyed, and their species, number of individuals, height, and DBH were recorded. At the same time, the habitat characteristics such as longitude, latitude, altitude and soil type of each plot were recorded (**Table 2**). Trees and shrubs in the plot were divided according to the biological characteristics of species.

**Table 2:** Basic situation of sample plot.

Plot	Succession time	Longitude	Latitude	Altitude/m	Soil type	Slope (°)	Slope aspect
Sha Jiyang	30a	118°39´04.43″	26°54´22.87″	355	Red soil	13	Northwest
Sha Jiyang	40a	118°39´65.03″	26°53´69.93″	422	Red soil	23	west
Huang Liyuan	50a	118°37´46.13″	26°55´61.63″	281	Red soil	20	Southwest
Shuang kenkou	70a	118°38´12.05″	26°55´93.66″	289	Red soil	12	west

### 2.3 Species importance value

The species importance value is a quantitative indicator that represents the status and role of a species within a forest community. It is calculated via the following formula (Brown and Curtis, 1952):


IV=(relative frequency+relative density+relative dominance)/3

### 2.4 Stand volume

The forest stand volume represents the cumulative volume of all individual trees within a given stand. The formula for this calculation is as follows (Fujian Provincial Standard, 2019):


$$\begin{array}{c} Pinus\;massoniana\text{ binary volume model}:\\ \text{V}=0.000070728{\text{D}}^{1.874518}{\text{H}}^{0.908949} \end{array}$$

$$\begin{array}{c} \text{Binary tree volume model of broad-leaved trees}:\\ \text{V}=0.0000685634{\text{D}}^{1.933221}{\text{H}}^{0.867885} \end{array}$$

M=V/0.25 ha

where M is the stand volume; V is the volume of standing wood (cm^3^); D is the diameter at breast height (cm); and H is the tree height (m).

### 2.5 Spearman rank correlation coefficient

The Spearman rank correlation coefficient measures the correlation between two sets of variables by examining the differences in their ranks. The formula for calculating the Spearman rank correlation coefficient is as follows (Bishara and Hittner, 2012):


$${\text{r}}_{\text{(i,k)}}=1-\left[6\sum_{j-1}^{\text{N}}{\left({\text{X}}_{\text{ij}}-\bar{{\text{X}}_{\text{i}}}\right)}^{2}\left({\text{X}}_{\text{kj}}-\bar{{\text{X}}_{\text{K}}}\right)\right]/\left({\text{N}}^{3}\text{-N}\right)$$

where ${\text{r}}_{\text{(i,k)}}$ represents the Spearman rank correlation coefficient between species i and species j in a sample plot, with a value range of [-1, 1]; N represents the total number of sample plots; X_ij_ and X_kj_ represent the ranks of species i and k in a quadrat, respectively; and ( $\bar{{\text{X}}_{\text{i}}}$ ) and ( $\bar{{\text{X}}_{\text{K}}}$ ) represent the average abundance of species i and k in all quadrats across all sample plots, respectively.

### 2.6 Biodiversity index

$\text{Margalef}\left({\text{d}}_{\text{Mα}}\right):\text{R}=\left(\text{S}-1\right)\ln\text{N}$


$$\text{Simpson}\left(\text{D}\right):\text{λ}=1-\sum^{}{\text{P}}_{i}^{2}$$

$$\text{Shannon}\left({\text{H}}_{e}^{'}\right):\text{H}=-\sum^{}{\text{P}}_{\text{i}}\ln{\text{P}}_{\text{i}}$$

$$\text{Pielou}\left({\text{J}}_{\text{e}}\right):\text{Jsw}=-\sum^{}\left({\text{P}}_{\text{i}}\ln{\text{P}}_{\text{i}}\right)/\ln\text{S}$$

where S represents the total number of species, N represents the total number of individuals across all species, P_i_ represents the proportion of individuals of species i (Sun, 2010).

## 3 Results

### 3.1 Species richness and importance values across different successional stages

**Figure 2:**
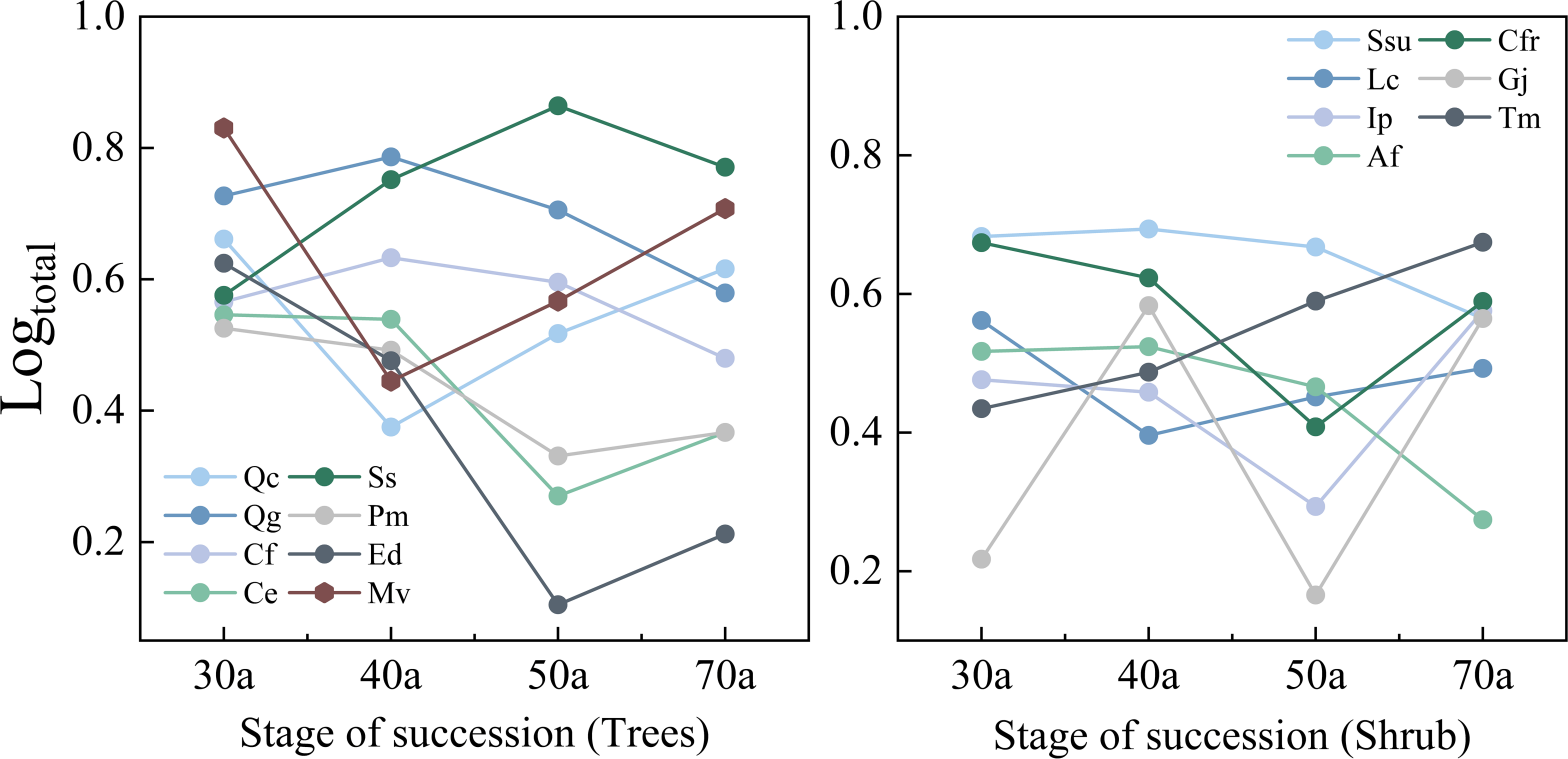
Changes in the relative numbers of different trees and shrubs during community succession. Ip, *Ilex pubescens*; Af, *Alniphyllum fortunei*; Gj, *Gardenia jasminoides*. The abbreviations of the other tree species in the figure are listed in **Table 1**. The same applies below.

**Table 3** and **Figure 2** show the variations in the importance values and relative abundances of the dominant species across the different successional stages. The 30-year-old community present relatively low interspecific differences in importance values; shrubs are dominant in terms of abundance, whereas trees are dominant in terms of size. In the 40-year-old community, interspecific differences in importance values increase, and trees such as *Q. glauca*, *S. superba*, *C. fargesii*, and *C. eyrei* are most plentiful, while the relative abundance of shrubs decreases. The 50-year-old community presents the greatest interspecific differences in importance values, with some trees experiencing a decline in relative abundance. *S. superba* achieves the highest importance value. As succession progresses to 70 years, the interspecific differences in importance values decrease. The relative abundances of both *Q. chungii* and *Q. glauca* declines, but their importance values remain high because of their size advantage. *S. superba*, despite its relatively high abundance, lacks a size advantage and therefore has a lower importance value than *Q. chungii*, *Q. glauca*, and *C. eyrei*. Additionally, with the progression of succession, the relative abundance differences among tree species increase and peak at 50 years. However, the relative abundance of shrub species does not clearly change with succession.

**Table 3:** Important values of the dominant species in the forest community at different succession times.

Succession time	Sort	Species name	Abbreviation	Important values
30a	1	*Machilus velutina*	Mv	10.2
	2	*Quercus glauca*	Qg	9.4
	3	*Quercus chungii*	Qc	8.8
	4	*Castanopsis fargesii*	Cf	5.7
	5	*Castanopsis eyrei*	Ce	4.9
	6	*Schima superba*	Ss	4.5
	7	*Elaeocarpus decipiens*	Ed	4.1
	8	*Symplocos sumuntia*	Ssu	4.1
	9	*Lithocarpus glaber*	Lg	4.1
	10	*Camellia fraterna*	Cfr	3.7
40a	1	*Quercus glauca*	Qg	18.9
	2	*Schima superba*	Ss	11.7
	3	*Castanopsis fargesii*	Cf	10.3
	4	*Castanopsis eyrei*	Ce	5.2
	5	*Symplocos sumuntia*	Ssu	4.7
	6	*Camellia fraterna*	Cfr	4.3
	7	*Machilus velutina*	Mv	3.8
	8	*Elaeocarpus decipiens*	Ed	3.6
	9	*Eurya nitida*	En	3.6
	10	*Symplocos sumuntia*	Ssu	3.4
50a	1	*Schima superba*	Ss	22.2
	2	*Quercus glauca*	Qg	19.3
	3	*Castanopsis fargesii*	Cf	12.4
	4	*Symplocos sumuntia*	Ssu	5.6
	5	*Tarenna mollissima*	Tm	5.0
	6	*Quercus chungii*	Qc	4.6
	7	*Machilus velutina*	Mv	4.4
	8	*Pinus massoniana*	Pm	4.2
	9	*Symplocos sumuntia*	Ssu	3.0
	10	*Loropetalum chinense*	Lc	2.7
70a	1	*Quercus chungii*	Qc	10.9
	2	*Quercus glauca*	Gg	9.6
	3	*Castanopsis fargesii*	Cf	9.2
	4	*Schima superba*	Ss	9.0
	5	*Pinus massoniana*	Pm	6.9
	6	*Tarenna mollissima*	Tm	4.6
	7	*Castanopsis eyrei*	Ce	4.6
	8	*Machilus velutina*	Mv	6.1
	9	*Itea chinensis*	Ic	3.2
	10	*Adinandra millettii*	Am	3.1

### 3.2 Stand volume, density, and diameter class distribution

**Figure 3:**
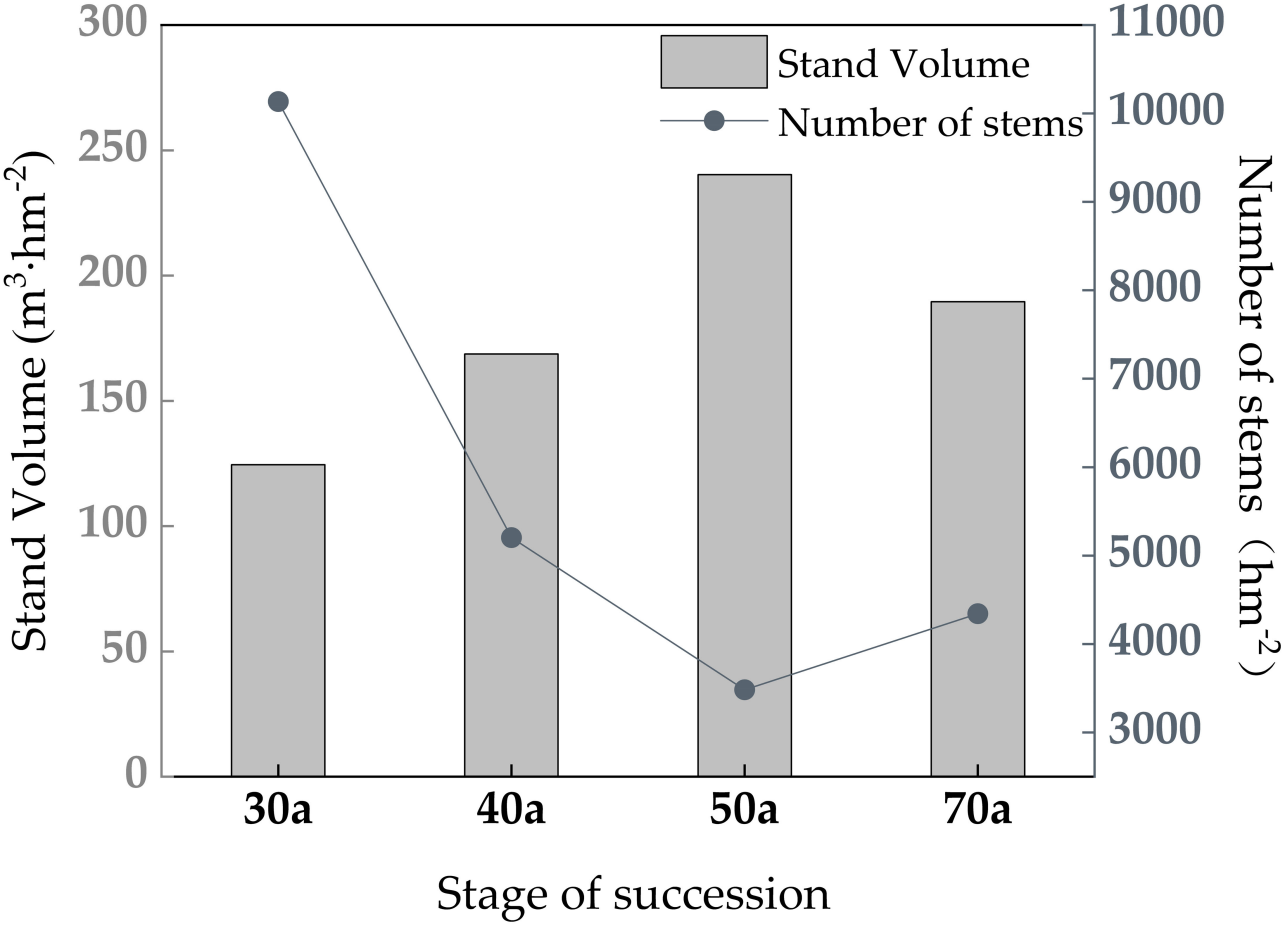
Changes in stand volume and density at different succession times.

In the process of community succession from 30-70-years, the stand volume increased first and then decreased, and the density change showed the opposite trend with the stand volume (**Figure 3**). During the 30-50-year succession period, the forest stand volume tends to increase, which is concurrent with a decrease in density. This process suggests a pronounced dominance of large trees in the 50-year-old com-munity, which, despite having the lowest density, achieves the highest volume. In the latter stage of succession from 50 to 70 years, an increase in density is observed along-side a decrease in volume, indicating the gradual replacement of large trees by medium-and small-diameter trees.

**Figure 4:**
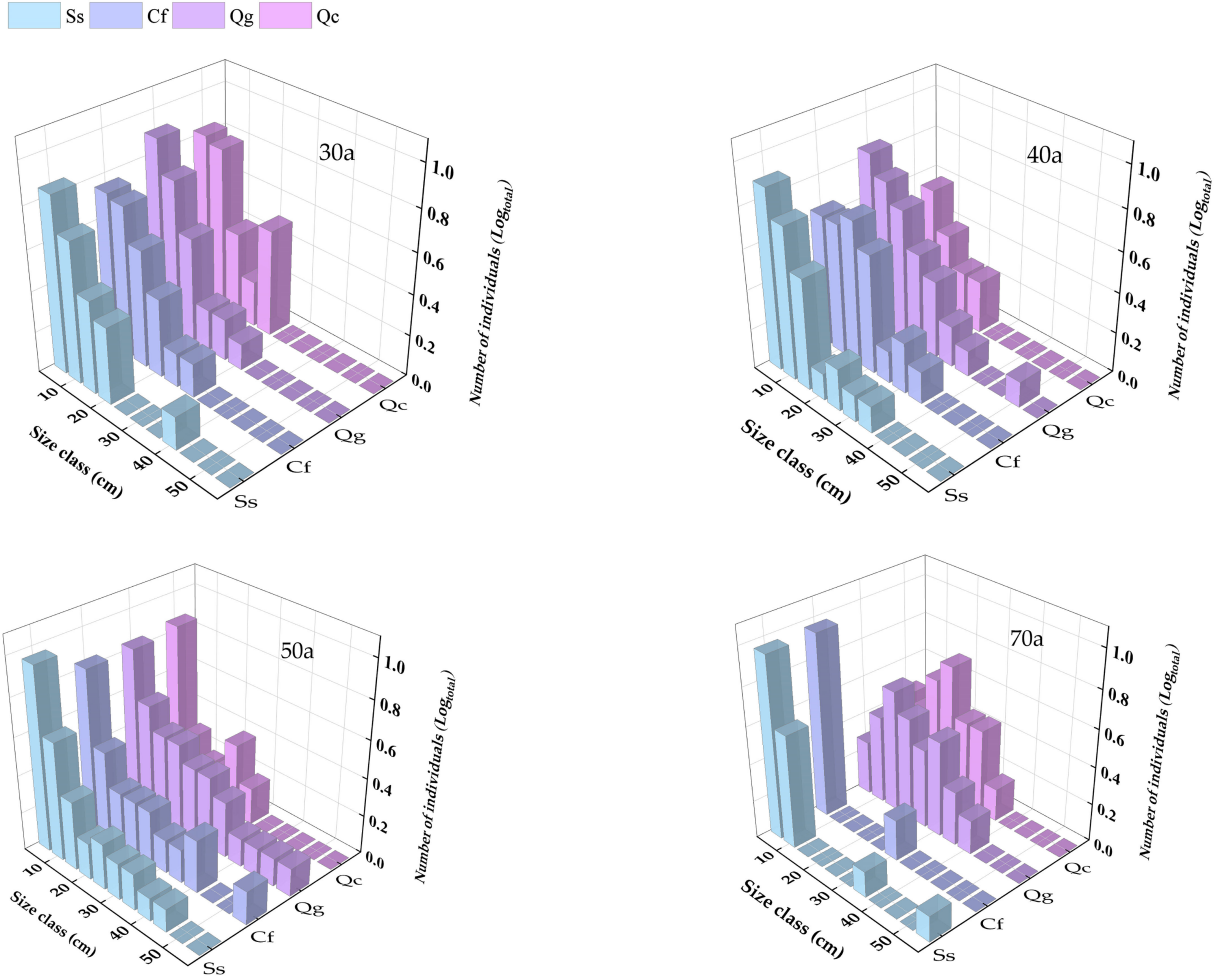
Changes in the diameter class distributions of the four dominant tree species at different successional times.

As shown in **Figure 4**, In the process of community succession from 30 years to 70 years, the DBH of trees increased gradually, and the number of trees increased first and then decreased. In the 30-year-old successional community, all four species present a reverse “J”-shaped distribution of DBH, with the majority of trees falling within the 5-25 cm range. As succession progresses, larger trees with DBHs > 30 cm begin to appear in the 40-year-old community. By 50 years, the relative abundance of trees with DBHs in the 30-50 cm range increases significantly, although trees with DBHs of 5-15 cm remain the most numerous. In the 70-year-old community, the relative abundance of small-diameter trees (DBH 5-10 cm) is highest for *S. superba* and *C. fargesii* and still displays a reverse “J”-shaped distribution, indicating the presence of abundant juvenile regeneration. In contrast, *Q. chungii* and *Q. glauca* present bell-shaped distributions, with the highest number of trees falling within the 15-30 cm DBH range.

### 3.3 Interspecific correlations and biodiversity

**Figure 5:**
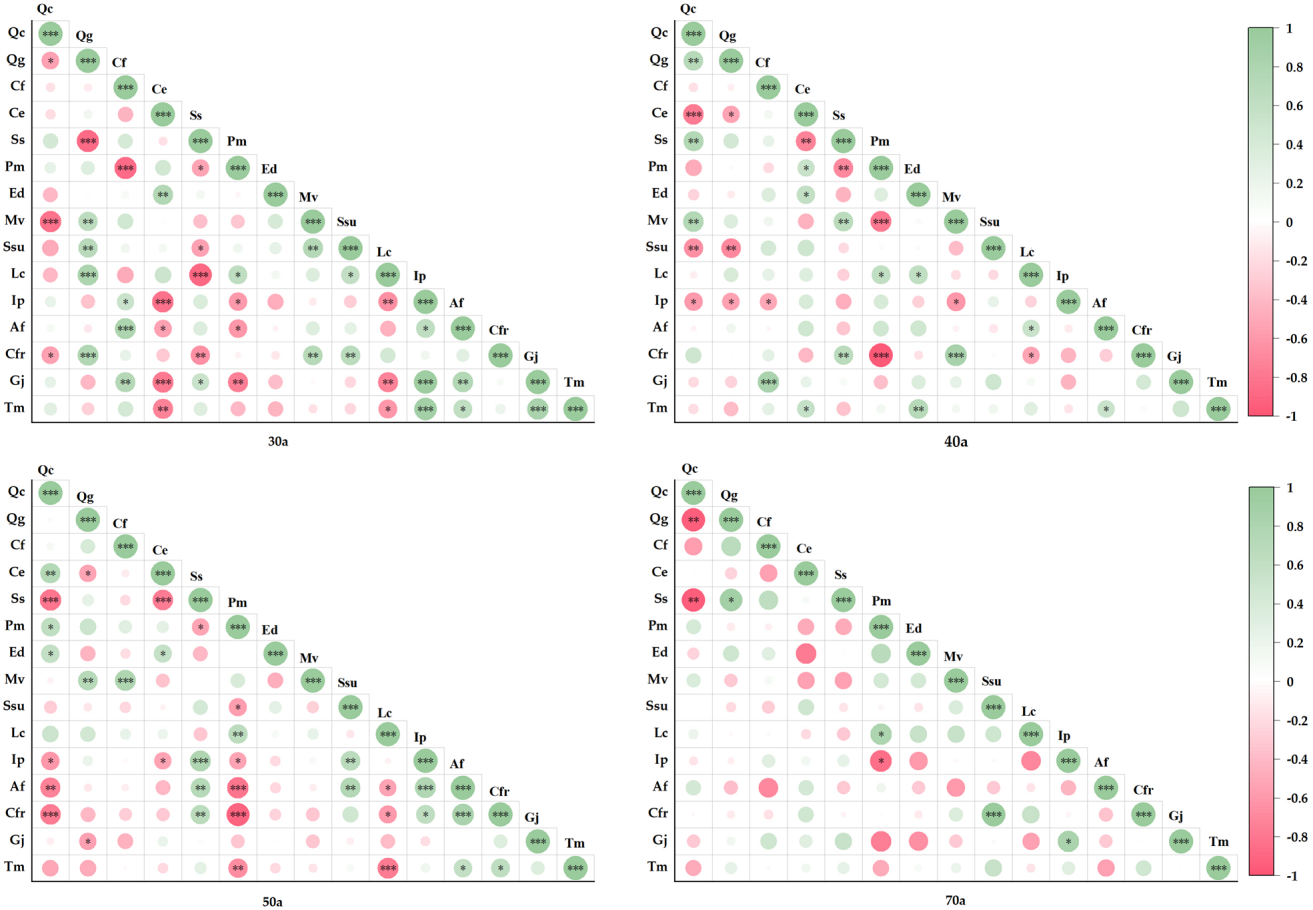
Semimatrix of the Spearman rank correlation coefficient among the main species in different successional stages (*P<=0.05, **P<=0.01, ***P<=0.001).

**Figure 5** shows the species pairwise correlations in communities at different stages of succession (30, 40, 50, and 70 years). Positive and negative correlations accounted for 47.6% and 52.4% of the total pairwise correlations in the 30-year-old community, respectively. Among these, 19 pairs presented significant positive correlations, 19 pairs presented significant negative correlations, and 63.8% were not statistically significant. In the 40-year-old community, positive and negative correlations accounted for 48.6% and 51.4%, respectively. Among these, 15 pairs presented significant positive correlations, 13 pairs presented significant negative correlations, and 73.3% were not statistically significant. In the 50-year-old community, positive and negative correlations accounted for 57.1% and 42.9%, respectively. Among these, 17 pairs presented significant positive correlations, 17 pairs presented significant negative correlations, and 67.6% were not statistically significant. Finally, in the 70-year-old community, positive and negative correlations accounted for 59.1% and 40.9%, respectively. Among these, only 4 pairs presented significant positive correlations, 3 pairs presented significant negative correlations, and 93.3% were not statistically significant.

**Figure 6:**
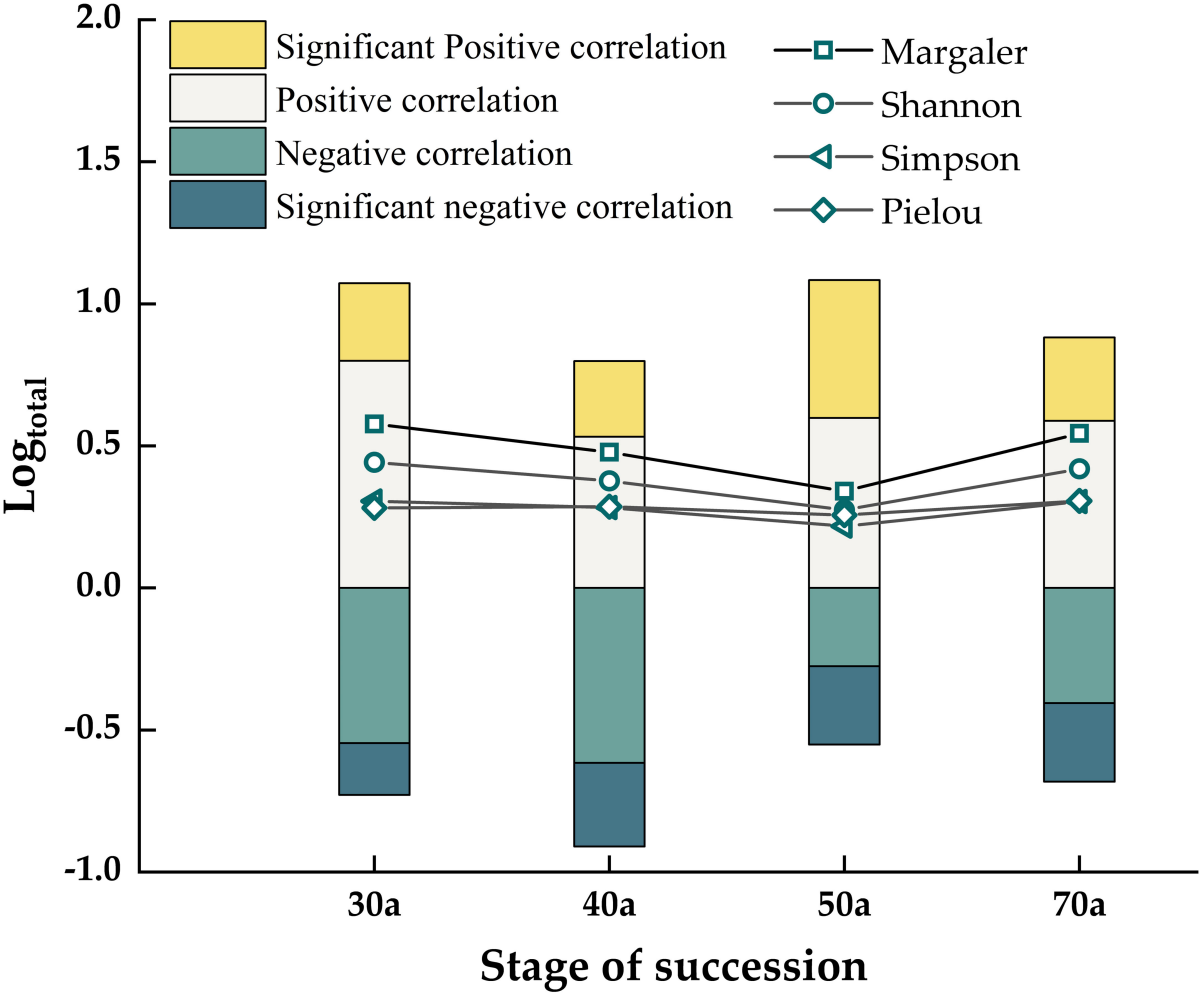
Changes in biodiversity and correlations.

**Figure 6** shows that in the 30-70-year succession process of the Fagaceae community, the diversity index of each species decreased first and then increased. Notably, the dMα, ${\text{H}}_{e}^{'}$ , D, and J_e_ indices for the 50-year-old community were significantly lower than those for the 30-, 40-, and 70-year-old communities (*P*< 0.05). The 30 a community had a significant positive correlation and high biodiversity. By 40 a, the significant negative correlation of the community tended to be dominant, and the biodiversity was slightly reduced. The 50 a positive correlation increased, and the biodiversity was the lowest. The community was in the 70 a stage, and the biodiversity gradually increased (**Table 4**).

**Table 4:** Forest species diversity indices at different stages of succession.

Stage of succession	Margaler	Shannon	Simpson	Pielou
30 a	4.88 ± 0.70 a	2.70 ± 0.21 a	0.90 ± 0.03 a	0.86 ± 0.04 b
40 a	3.17 ± 0.77 b	2.12 ± 0.31 b	0.84 ± 0.06 b	0.90 ± 0.04 a
50 a	2.55 ± 0.68 c	1.85 ± 0.35 c	0.76 ± 0.13 c	0.82 ± 0.09 c
70 a	4.46 ± 1.01 a	2.57 ± 0.31 a	0.90 ± 0.05 a	0.90 ± 0.05 a

Significant differences between different successional stages are represented by lowercase letters (*P*< 0.05).

## 4 Discussion

### 4.1 Dynamics of dominant tree species and stand structures across successional stages

Our study revealed that, in the early stages of succession, the importance values of the dominant tree species were relatively similar. However, in the middle and late stages of succession, the importance values of shade-tolerant species such as *Q. chungii* and *Q. glauca* increased, and the relative abundance of tree species in the canopy layer also gradually diverged. This finding aligns with previous research conducted in secondary forests in northern Guangdong and the Changbai Mountains (Zhang et al., 2019; Chen et al., 2022). During the early stages of succession, the dense distribution of trees and shrubs results in minimal differences among the dominant species. In the middle and late stages, neutral and shade-tolerant species are more advantageous in terms of light and nutrient competition, allowing them to establish canopy dominance (Chou et al., 2018). However, research has shown that in frequently disturbed secondary forests, such as those undergoing selective cutting or recovering from fire, the recovery stage often features dominant populations of sun-loving species. This is because disturbances alter the light conditions within the forest, favoring the regeneration of sun-loving species (Liu et al., 2021; Lan and Liu, 2022). Thus, the emergence of dominant species is influenced not only by interspecific competition but also by environmental and anthropogenic factors that can alter species distribution patterns and functional traits, leading to different patterns of succession (Chesson, 1994; Cain and Shelton, 2001). With ongoing changes in the global climate, previous studies have reported a decline in populations of Fagaceae species, including Fagus spp (Wang et al., 2012). For example, due to the increase of summer temperature in European beech, the seed yield is reduced and the plant has a mast phenomenon (Foest et al., 2024). However, whether similar trends affect the Cyclobalanopsis- and Castanopsis-dominated communities in our study area remains to be investigated.

The subtropical Fagaceae communities in our study exhibited typical metapopulation characteristics, with populations of *Q. chungii* and *Q. glauca* experiencing minimal fluctuations, whereas populations of *S. superba* and *C. fargesii*. exhibited greater fluctuations. However, it remains unclear whether these communities are in long-term dynamic equilibrium, necessitating further research involving long-term observations or larger spatial scales.

Additionally, during the 30 a - 50 a successional stage, the forest stand volume in-creased rapidly despite a decrease in stand density. As forests develop, trees begin to compete for limited resources such as sunlight, water, and soil nutrients; these factors affect population size and individual growth and often lead to self-thinning phenomena in dense stands (Li, 2002). In contrast, an appropriate reduction in density allows plants to access sufficient nutrient space, promoting succession towards stands with larger diameter trees and greater volume (Fernández-Tschieder and Binkley, 2018; Farooq et al., 2021). This study also revealed that in the early stages of succession, the diameter class distribution of most tree species exhibited an inverted “J” shape, indicating the presence of abundant resources for seedling growth. At 40 a-50 a successional stage, the main tree species shifted towards larger diameter classes. In the later stages of succession, large trees such as *Q. chungii* and *Q. glauca* dominated the canopy, whereas *S. superba* and *C. fargesii* experienced a decline in large-diameter trees. This change was attributed to intense competition for canopy light resources among the dominant species, leading to individual mortality. However, as forest gaps formed, the understory environment changed, particularly with respect to light intensity, quality, and duration, favoring the regeneration of *S. superba* and *C. fargesii* within the gaps (Coates, 2002). This study explored the dynamic changes of dominant tree species and population size in different successional stages of the subtropical Fagaceae community. However, this study was limited to density constraints, and lacked discussion on negative density constraints and non-density constraints. In the future, the community structure model can be improved by combining remote sensing technology, GIS and other modern technical means on the basis of long-term community monitoring, so as to predict and manage the future development of forest ecosystem more accurately.

### 4.2 Dynamic changes of interspecific relationship

In this study, the interspecific relationship of the Fagaceae community was analyzed. It was discovered that in the 30 a - 40 a succession process of the community, the negative correlation between species pairs accounts for a relatively high proportion. This is similar to the results of Lv et al. (2019) on the dominant tree species of natural forests in North China Reserve, which may be due to the fierce competition for space and resources among community species. Wang et al. (2024) studied the community of Chinese fir plantation in Jianfengling, Hainan, and found that the overall association of the community showed a significant positive association, which was inconsistent with the results of this study, possibly due to differences in latitude changes. At 50 a - 70a, the positive correlation ratio of community species increased, and neutral and shade-tolerant (*S. superba*, *Q. glauca, Q. chungii*, etc.) species gradually dominated. At the same time, there was a negative correlation between *P. massoniana* and *Q. glauca*, *C. fargesii* and *Q. chungii* in 70 a, indicating that there was a competitive relationship between species, but it was worth noting that *P. massoniana* and *C. fargesii* saplings could still exist in the community, which was in line with competitive coexistence (Ecological Terminology Approval Committee, 2006). Simultaneously, this study found that at 70 a, the species maintained a certain degree of independence, which is consistent with the results of the study of *Loropetalum chinense* community in Guilin Karst Rocky Mountain by Jian et al. (2021), indicating that the community can form a self-regulation mechanism in the recovery stage and maintain a relative dynamic balance. However, the interspecific relationship only reveals the interspecific dynamic changes during the succession of Fagaceae community, and does not consider the influencing factors such as allelopathy, natural environment and geographical location. Therefore, in the future, the internal mechanism of species coexistence and competition should be deeply analyzed from the aspects of molecular biology, ecological geography and plant physiology. At the same time, it is necessary to strengthen the research on the self-regulation mechanism within the community, such as forest gap dynamics, seedling regeneration and other processes, which will help to deeply analyze the ecological mechanism of the formation of interspecific relationships.

### 4.3 Interspecific relationships and biodiversity

Our study revealed that species diversity initially decreased but then increased. Some researchers argue that species diversity gradually increases with community succession (Zhang et al., 2020), contradicting our findings, possibly influenced by site conditions and vegetation types. Howard and Lee (2003) reported that species richness in New England forest communities gradually declined with succession, similar to our results, thus supporting the “initial floristic composition” model (Egler, 1954), which suggests that all species exist at the beginning of succession and that the process involves a selective elimination scheme. One possible reason for this result is related to interspecific relationships. As forests develop, species may be replaced or disappear. In the present study, in the early stages of succession, a high proportion of significant positive correlations and the highest diversity index indicate that shrubs and trees co-exist and thrive. However, during the 30- 50 a period, the emergence of large trees led to the disappearance or replacement of some species due to resource scarcity, causing a decline in the diversity index and an increase in negative correlations. Dominant tree species and their associated species occupied advantageous positions in the competition for survival. At 50 a- 70 a, the diversity index increased due to the supplementation of seedlings, and positive correlations also increased. These results align with the intermediate disturbance hypothesis (Connell, 1978), which suggests that community diversity can be enhanced under moderate disturbance. During succession, plants compete to increase resource availability, thereby changing the function and characteristics of ecosystems. Moderate disturbances during succession are beneficial for maintaining community diversity and stability (Callaway, 2007; Catford et al., 2012). The study found that the interspecific relationship changed from positive correlation to negative correlation, and then to positive correlation. During this period, species diversity did not gradually increase with succession, but showed a trend of decreasing first and then increasing, indicating that interspecific competition and symbiotic relationship had an important impact on species diversity during succession. In addition, the competition of resources such as light, water, nutrients and space is an important factor affecting the relationship between interspecific relationship and biodiversity. Therefore, in the future, based on the combination of long-term dynamic monitoring plots and environmental factors, the subtropical crustacean community and even other communities can be studied to reveal the relationship between interspecific relationship and biodiversity at different succession stages and the potential ecological mechanism.

## 5 Conclusions

In summary, The difference of important value of 30 a- 40 a community is small, the differentiation of forest is not obvious, the diameter class structure is inverted J type distribution, and the negative correlation between species is relatively high. With the succession, the difference of important value of 50 a community became larger, the differentiation of forest trees was obvious, the diameter class structure showed an inverted J-shaped distribution, and the trees with DBH above 30 cm appeared. At this time, the interspecific relationship with the highest volume was mainly positive correlation, and the biodiversity decreased. By 70 a, the difference of importance value between tree species became smaller, the degree of forest differentiation was high, the diameter structure of shade-tolerant tree species changed to bell-shaped distribution, and the insignificant correlation between species accounted for a large proportion, reflecting strong independence, biodiversity increased, and the community maintained a relative dynamic balance. The results of this study will contribute to understanding the changes in community structure, interspecific relationship, and community development status of Fagaceae, and provide a theoretical basis for further exploration of subtropical forest construction, vegetation restoration, and reconstruction. Currently, this study only analyzes the community structure and interspecific relationship of Fagaceae in subtropical Fujian, and the results are relatively one - sided. Future research could integrate subtropical forests from various regions to obtain comprehensive data and disclose its universal laws. In addition, the secondary forest of Fagaceae community is widely distributed in subtropical forests in southern China. In the timber forest area, it is recommended to carry out sanitary cutting and transparent cutting measures in the secondary forest before 30 a to promote the growth and development of *Q. glauca*, *Q. chungii*, *S. superba* and other economically valuable trees in Fujian. After 30 a, the growth cutting measures should be implemented to promote the DBH growth of the stand and reach the ideal harvest state at 50 a, so as to realize the sustainable utilization of timber forest. In terms of public welfare forests, it is recommended not to use excessive human interference, monitor the health and integrity of forest regeneration and development once every 5 years, and carry out appropriate forest cleaning and replanting if necessary.

## Data Availability

The raw data supporting the conclusions of this article will be made available by the authors, without undue reservation.
